# Platelet Adhesion to Podoplanin Under Flow is Mediated by the Receptor CLEC-2 and Stabilised by Src/Syk-Dependent Platelet Signalling

**DOI:** 10.1160/TH14-09-0762

**Published:** 2015-02-19

**Authors:** Leyre Navarro-Núñez, Alice Y. Pollitt, Kate Lowe, Arusa Latif, Gerard B. Nash, Steve P. Watson

**Affiliations:** Centre for Cardiovascular Sciences, Institute for Biomedical Research, College of Medical and Dental Sciences, University of Birmingham, Birmingham, UK

**Keywords:** Platelet physiology, rheology, lymphatics, adhesion molecules, signal transduction

## Abstract

Platelet-specific deletion of CLEC-2, which signals through Src and Syk kinases, or global deletion of its ligand podoplanin results in blood-filled lymphatics during mouse development. Platelet-specific Syk deficiency phenocopies this defect, indicating that platelet activation is required for lymphatic development. In the present study, we investigated whether CLEC-2-podoplanin interactions could support platelet arrest from blood flow and whether platelet signalling is required for stable platelet adhesion to lymphatic endothelial cells (LECs) and recombinant podoplanin under flow. Perfusion of human or mouse blood over human LEC monolayers led to platelet adhesion and aggregation. Following αIIbβ3 blockade, individual platelets still adhered. Platelet binding occurred at venous but not arterial shear rates. There was no adhesion using CLEC-2-deficient blood or to vascular endothelial cells (which lack podoplanin). Perfusion of human blood over human Fc-podoplanin (hFcPDPN) in the presence of monoclonal antibody IV.3 to block FcγRIIA receptors led to platelet arrest at similar shear rates to those used on LECs. Src and Syk inhibitors significantly reduced global adhesion of human or mouse platelets to LECs and hFcPDPN. A similar result was seen using Syk-deficient mouse platelets. Reduced platelet adhesion was due to a decrease in the stability of binding. In conclusion, our data reveal that CLEC-2 is an adhesive receptor that supports platelet arrest to podoplanin under venous shear. Src/Syk-dependent signalling stabilises platelet adhesion to podoplanin, providing a possible molecular mechanism contributing to the lymphatic defects of Syk-deficient mice.

## Introduction

Beyond their well-known role as mediators of haemostasis and thrombosis, platelets regulate mouse embryonic lymphatic development. This process begins when Prox1-expressing lymphatic endothelial cell (LEC) progenitors start migrating and budding away from the cardinal vein and intersomitic vessels at around embryonic day (E) 10.0 (1, 2), soon after platelets are first detected in the yolk sac at E9.5(3). These LEC progenitors acquire extensive podoplanin expression once they have fully exited the cardinal vein by E11.5 and develop into lymphatic sacs and the primordial thoracic duct. The development of lymphovenous valves in the contact area between the podoplanin-expressing thoracic duct and the cardinal vein separates both structures, preventing venous backflow into the lower-pressure nascent lymphatics (1).

Mice deficient in the platelet C-type lectin receptor CLEC-2 or lacking essential components of its intracellular activation cascade – the tyrosine kinase Syk, adapter protein SLP-76 or effector enzyme PLCγ2 – develop a characteristic phenotype with blood-lymphatic mixing, oedema, absence of lymph nodes and brain haemorrhaging during mid-gestation (E12.5 onwards) (4–7). Podoplanin (PDPN), the only known endogenous ligand for CLEC-2, is expressed in various cell types including LECs but not vascular endothelial cells. Noteworthy, mice deficient in this glycoprotein (8, 9) or lacking the glycosyltransferase T-synthase critical for its biosynthesis (10) show similar developmental abnormalities as CLEC-2 or Syk-deficient mice. Together, these results suggest that binding of platelet CLEC-2 to podoplanin and subsequent platelet activation are both required for development of a number of tissues and organs, including the lymphatics. The description of blood-filled lymphatics in mice devoid of megakaryocytes/platelets (11) provides further evidence of their role in lymphatic development.

The presence of anomalous lymphatics after postnatal genetic deletion of podoplanin (10), or in CLEC-2– or Syk-deficient radiation chimeric mice (6, 12) reveals the essential role of these proteins in lymphatic repair and maintenance during adulthood. The importance of platelet CLEC-2 adhesive interactions as key mediators of adult lymphatic maintenance has been highlighted by the demonstration of platelet binding to LECs on the terminal portion of the mouse thoracic duct in contact with the subclavian vein, where conditions of low flow and low shear are expected (13, 14). Absence of CLEC-2-mediated platelet interactions in this lymphovenous connection following antibody-induced CLEC-2 depletion in circulating platelets leads to blood-filled lymphatics (14).

Additionally, recent reports have identified partially redundant functions for platelet CLEC-2 and GPVI in haemostasis and thrombosis (15, 16). Nonetheless, genetic deficiency or pharmacological inhibition of Syk do not associate with major effects on bleeding or arterial thrombosis (17, 18). These data indicate that the unexpected role of CLEC-2 in haemostasis and thrombosis does not rely on downstream signalling but on yet undefined CLEC-2 adhesive functions, although intravascular ligands for CLEC-2 have not been identified. Although platelet aggregate formation on LECs under flow has been reported *in vitro* (4, 19), a comprehensive study of the conditions allowing flowing platelets to arrest onto a podoplanin-expressing surface has not been reported. As a result, it is currently unknown whether podoplanin and CLEC-2 acting alone are sufficient to induce platelet binding to LECs, which shear conditions allow such interactions, and the role of platelet intracellular signalling in this adhesion process. We have therefore characterised the binding of platelets to LECs and recombinant podoplanin under various *in vitro* flow conditions and evaluated the requirement of intracellular signalling downstream of CLEC-2 for optimal platelet adhesion to podoplanin.

Our results indicate that direct adhesive interactions between CLEC-2 and podoplanin-expressing cells can occur at venous but not arterial shear rates, and demonstrate that such binding is stabilised by Src/Syk-dependent platelet signalling, which also mediates CLEC-2-dependent platelet aggregation. Our data reveal for the first time the adhesive properties of the receptor CLEC-2, provide a possible molecular mechanism contributing to the lymphatic defects observed in Syk-deficient mice, and carry important implications for the use of Src and Syk inhibitors in clinical settings.

## Material and methods

Detailed information of the reagents used and additional methods are included in the Suppl. Material available online at www.thrombosis-online.com).

### Mouse models

All procedures obtained United Kingdom Home Office approval (project licence number 30/2721). To study the physiological roles of CLEC-2 without the developmental defects and decreased platelet counts observed in the previously described PF4-Cre CLEC-2 mice (6), genetically modified C57Bl/6 mice bearing a floxed CLEC-2 allele (6, 20) were crossed with ROSA26^CreERT2^ animals (21) to obtain CLEC-2^floxed/floxed;^ ^CreERT2^ mice. The ROSA26^CreERT2^ mice carry a conditional Cre recombinase allele fused to an oestrogen receptor moiety targeted to the ubiquitously expressed ROSA26 locus. Tamoxifen administration induces Cre translocation to the nucleus permitting inducible recombination of LoxP sites. To induce excision of the floxed CLEC-2 allele, mice were treated with 100 µl tamoxifen (10 mg/ml in corn oil) or corn oil as control by intra-peritoneal injection once daily for five consecutive days. Complete loss of platelet CLEC-2 expression in the tamoxifen-treated mice was confirmed by flow cytometry on the day of the experiment.

Radiation chimeras were generated as described previously (22). Briefly, six-week-old C57Bl/6 mice were treated with Baytril for one week followed by irradiation with two doses of 500 rad, 3 hours (h) apart. Mice were then injected with 1.5 × 10^6^ Syk^**+/+**^ or Syk^**–/–**^ foetal liver cells (23). The genotype of reconstituting cells was confirmed by PCR. Mice were used for experimentation eight weeks post-transplantation.

### Platelet adhesion to endothelial cell monolayers under flow

Confluent human LECs or HUVECs were dissociated, seeded in Ibidi µ-slides VI (Ibidi GmbH, Martinsried, Germany) and cultured until a confluent monolayer was formed (usually 48 h).

Human blood from healthy volunteers that had not taken any medication affecting platelet function for at least 10 days was drawn after informed consent into 5 U/ml heparin and 40 µM PPACK as anticoagulants. Ethical approval was granted by the University of Birmingham Research Ethics Committee (reference UHSP/22/BTVR/07). Mouse blood was drawn from the vena cavae of terminally CO_2_-narcosed mice following isofluorane anaesthesia into the same anticoagulant mixture. Mouse platelet counts were measured using an ABX Pentra 60 Hematology Analyzer (Block Scientific, Bohemia, NY, USA).

Whole blood was pre-incubated with inhibitors at 37°C for 10 minutes (min) as appropriate and then perfused at 37°C over endothelial monolayers for 4–8 min at different wall shear rates representing venous (50, 150 s^-1^) and arterial shear rates (450–1350 s^-1^) (24) using a syringe pump (Harvard apparatus, Holliston, MA, USA). Slides were washed for 2 min at the same shear rate with modified Tyrode’s buffer (136 mM NaCl, 0.42 mM Na_2_HPO_4_, 2.7 mM KCl, 5 mM HEPES, 5.6 mM glucose, 2 mM MgCl_2_, 0.1% BSA; pH 7.4) before fixation with 3.7% formaldehyde.

Remaining fixative was quenched with 50 mM NH_4_Cl and slides stained at room temperature in the dark with anti-human podoplanin and anti-CD41 primary antibodies (2 µg/ml in 2% BSA-PBS) followed by appropriate secondary antibodies (1:1,000 in 2% BSA-PBS) as stated in the figure legends. Slides were then mounted with Ibidi mounting medium (Ibidi GmbH).

### Platelet adhesion to recombinant podoplanin under flow

Ibidi µ-slides VI were coated with human Fc-podoplanin fusion protein (hFcPDPN: 100 µg/ml in PBS, 1 h at room temperature), washed and blocked with 5 mg/ml fatty acid-free BSA in PBS. Human or mouse blood drawn as explained above was treated with the fluorescent dye DiOC6 (5 µg/ml) and inhibitors when appropriate and perfused at 37 °C through the hFcPDPN coated Ibidi µ-slides at the stated shear rates. Channels were PBS-washed, formalin-fixed and mounted.

### Imaging

Immunofluorescence images were taken with a 63× oil immersion 1.40 NA objective in a Leica SP2 confocal microscope with sequential scanning for FITC or Alexa 488 (green) and PE or Alexa 568 (red). Platelet channel images (maximum intensity projections) were analysed off-line to assess platelet coverage using ImageJ (25) after segmenting greyscale images into binary data.

Phase contrast images were captured using a Zeiss LD Plan-Neofluar 20x/0.4 Ph2 lens on a Axiovert 200 inverted microscope (Zeiss, Welwyn Garden City, UK) and a Hamamatsu Orca 285 digital camera using Slidebook software (Intelligent Imaging Innovations GmbH, Göttingen, Germany).

### Analysis of platelet adhesion dynamics using time-lapse video microscopy

Dynamics of platelet adhesion to podoplanin were studied using a 20× objective lens in an inverted fluorescence microscope (Olympus IX70) fitted with a heated stage and a computerised digital camera using Image-Pro Plus software (DataCell Ltd, Finchampstead, UK). Videomicroscopic recordings were analysed off-line using ImageJ. Platelet binding stability was measured as percentage of adhesion events lasting at least 10 seconds (s).

To analyse real-time adhesion of platelets to LECs, heparinised whole blood was incubated with FITC-conjugated anti-human CD41 and CD42b antibodies at 1:100 dilution and inhibitors or vehicle control for 10 min and perfused over LEC monolayers at 50 s^-1^. Real-time adhesion of platelets to hFcPDPN was analysed in blood incubated with 4 µM DiOC6, 10 µg/ml IV.3 antibody and inhibitors or vehicle control for 10 min and perfused over hFcPDPN-coated channels at 50 s^-1^. Digital images were captured at intervals of 500 ms (LECs) or 250 ms (hFcPDPN) using 2×2 binning to create 1-min videos.

### Data analysis

Platelet adhesion results are expressed as percentage of surface area covered by platelets. Results are expressed as mean values ± standard error of the mean (SEM). Student’s t-test or one-way ANOVA statistical test, followed by Dunnett’s post-test where appropriate, were used as indicated in the figure legends. Differences with p values <0.05 were considered statistically significant.

## Results

### Lymphatic endothelial cells support platelet adhesion and aggregation at venous shear rates

**Figure 1:**
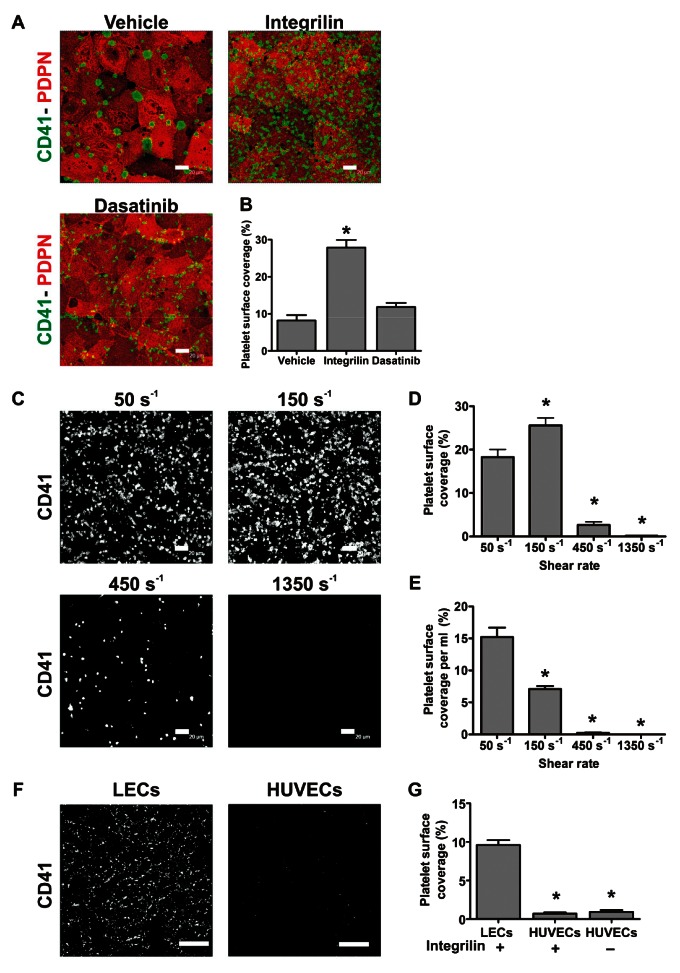
**LECs support human platelet adhesion and aggregation at venous shear rates.** A) Confluent human LECs were perfused with human blood for 8 min at 50 s^-1^ after treatment with vehicle (DMSO), 9 µM Integrilin or 10 µM dasatinib. Slides were immunostained with anti-podoplanin (PDPN)/anti-rat Alexa-568 and anti-CD41/anti-mouse Alexa-488 antibodies. Representative pictures are shown, n=3. Scale bars: 20 µm. B) Platelet surface coverage analysed off-line plotted as mean ± SEM. *p<0.05 (significant differences vs vehicle after one-way ANOVA and Dunnett’s post-test). C) Integrilin-treated blood was perfused for 4 min at the indicated shear rates over confluent LECs. Samples were stained with anti-CD41 plus anti-mouse Alexa-488 antibodies. Representative pictures are shown. LEC monolayer integrity was confirmed by podoplanin immunostaining. Scale bars: 20 µm. D, E) Percentage of absolute platelet surface coverage (D) and platelet coverage per ml of blood perfused (E), mean ± SEM, n=3. One-way ANOVA indicated a significant effect of shear on platelet coverage per ml perfused (p<0.0001). *p<0.05 (significant differences versus adhesion at 50 s^-1^ by Dunnett’s post-test). F) Confluent human LECs or HUVECs were perfused for 4 min at 50 s^-1^ with Integrilin-treated blood and stained with anti-CD41-FITC antibody. LEC monolayer integrity was confirmed by podoplanin immunostaining. Representative pictures are shown. Scale bars: 75 µm. G) Platelet surface coverage plotted as mean ± SEM, n=3. *p<0.05 (significant differences vs binding to LECs by one-way ANOVA with Dunnett’s post-test).

We first tested the ability of podoplanin expressed in human LEC monolayers to induce platelet arrest at a wall shear rate of 50 s^-1^. Perfusion of human blood over LEC monolayers for 8 min led to significant platelet adhesion, activation and subsequent aggregation (►Figure 1A). As previously shown using Born-aggregometry (6), LEC-induced aggregate formation was inhibited by the αIIbβ3 blocker eptifibatide (Integrilin). In the presence of this inhibitor, many individual platelets could still be seen attached to LECs. Abrogation of platelet-platelet interactions by Integrilin increased overall surface coverage (►Figure 1B).

Next, we compared the platelet coverage reached after 4 min perfusion at different shear rates: 50, 150, 450 and 1,350 s^-1^ (►Figure 1C). Human platelets adhered to LECs in great numbers at shear rates of 50 or 150 s^-1^, while adhesion was reduced at 450 s^-1^ and essentially absent at 1,350 s^-1^ (►Figure 1D). When adhesion was normalised as percentage of coverage per ml of blood perfused, the efficiency of platelet recruitment onto the LECs decreased steadily with increasing shear rate (►Figure 1E). Measurement of whole blood viscosity at 37 °C in microslides gave values of 3–4.5 mPa·s in this range of shear rates (data not shown) revealing that the wall shear stress allowing efficient platelet adhesion was <1 Pa. These data suggest that stable binding of platelets to LECs only occurs at levels of shear found in the venous circulation (24).

To test whether platelet binding was restricted to endothelial cells of lymphatic origin, we perfused whole blood at 50 s^-1^ over confluent monolayers of human umbilical vein endothelial cells (HUVECs, ►Figure 1F). Absence of podoplanin in these cells was confirmed by flow cytometry (Suppl. Figure 1, available online at www.thrombosis-online.com). We observed scarce platelets bound to HUVECs, either in the presence or absence of Integrilin (►Figure 1G). These results strongly indicate that the presence of podoplanin is required for platelet binding to endothelial cells under flow.

We then investigated the potential contribution of other endothelial adhesion molecules. CD31 (PECAM-1), expressed in LEC junctions and also in platelets (26), mediates homophilic intercellular adhesion events. Von Willebrand factor immobilised onto endothelial αvβ3 interacts *in vitro* with platelet glycoprotein Ib-IX-V under flow (27). To establish whether endothelial CD31 and β3 integrin (CD61) facilitate platelet binding to LECs, human LEC monolayers were treated with blocking antibodies before perfusing human whole blood for 8 min at 50 s^-1^. Platelet adhesion was then compared to that observed using an irrelevant IgG antibody (Suppl. Figure 2, available online at www.thrombosis-online.com). Our results did not reveal a significant effect of these antibodies, suggesting that CD31 and CD61 do not facilitate platelet-LEC interactions.

### Recombinant human podoplanin and LECs support similar platelet adhesion and aggregation

To further investigate whether podoplanin was responsible for the interactions between platelets and LECs, we tested whether purified human recombinant Fc-podoplanin (hFcPDPN) was sufficient to support platelet binding under flow. Since human platelets express the low affinity immunoglobulin receptor FcγRIIa (CD32a), these assays were performed in the presence of 10 µg/ml CD32 blocking antibody (clone IV.3) (28).

**Figure 2:**
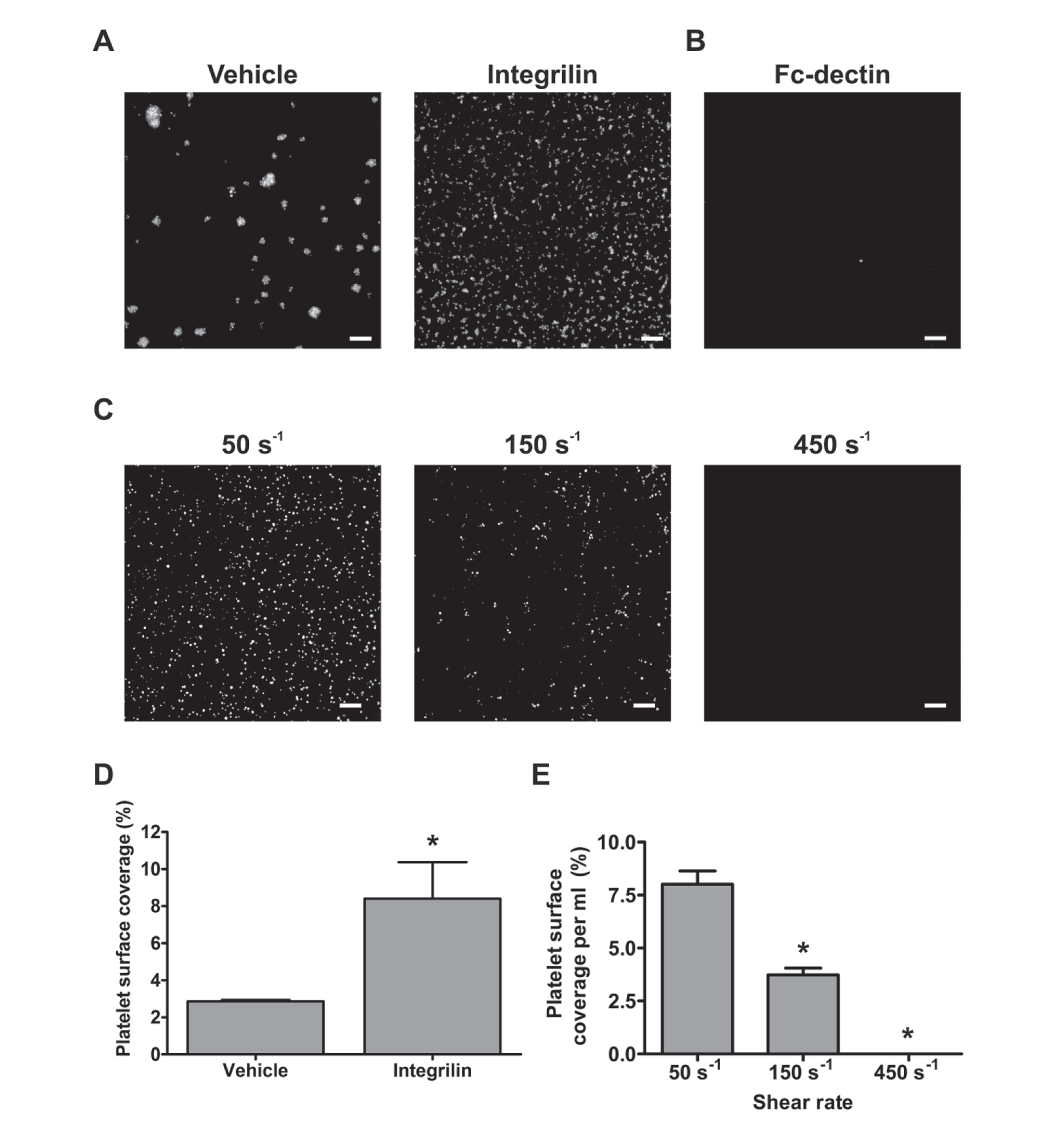
**Recombinant hFcPDPN supports similar platelet adhesion and aggregation to LECs.** A) Channels were coated with 100 µg/ml hFcPDPN and blocked with BSA. Heparinised blood treated with 5 µg/ml DiOC6 and 10 µg/ml IV.3 antibody was perfused for 4 min at 50 s^-1^ with vehicle (DMSO) or 9 µM Integrilin. B) Channels were similarly coated with 100 µg/ml hFc-dectin and perfused in the absence of IV.3 antibody with DiOC6-treated blood at 50 s^-1^. C) hFcPDPN-coated channels were perfused with blood treated as above at the stated shear rates in the presence of Integrilin. D) Quantification of platelet binding to hFcPDPN in the presence of vehicle or Integrilin plotted as mean ± SEM (n=3). *p<0.05 (significant differences vs vehicle-treated blood by two-tailed Student’s t-test). E) Evaluation of platelet binding at the different shear rates (mean ± SEM, n=3). One-way ANOVA indicated a significant effect of shear (p<0.0001). *p<0.05 (significant differences vs adhesion at 50 s^-1^ by Dunnett’s post-test). Scale bars: 20 µm.

Perfusion of whole blood at 50 s^-1^ over hFcPDPN-coated channels led to platelet adhesion and aggregation. In the presence of Integrilin, deposition of numerous individual platelets could be seen (►Figure 2A, D). Recombinant Fc-dectin – which belongs to the same family as CLEC-2 – or BSA did not induce platelet binding, even in the absence of IV.3 antibody (►Figure 2B and data not shown). We further investigated whether hFcPDPN supported platelet adhesion at the same shear rates as human LECs by perfusing whole blood through hFcPDPN-coated channels at 50, 150 and 450 s^-1^ under non-aggregating conditions (►Figure 2C). We observed that levels of platelet recruitment onto hFcPDPN or LECs similarly diminished with increasing shear rates, with very few platelets bound to hFcPDPN at 450 s^-1^ (compare ►Figure 1E and ►Figure 2E). These results reveal that podoplanin is sufficient to induce arrest and activation of flowing platelets.

### CLEC-2 mediates binding of mouse platelets to human recombinant podoplanin or human LECs under flow

**Figure 3:**
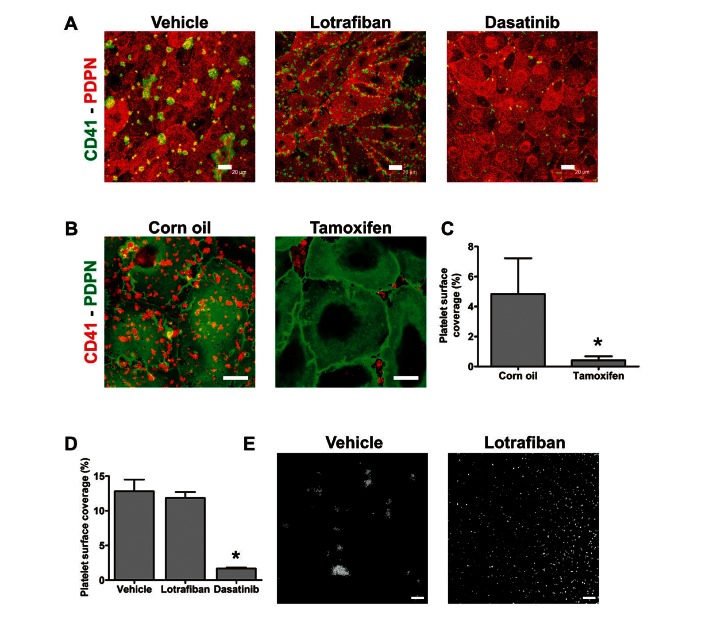
**CLEC-2 mediates binding of mouse platelets to human LECs or hFcPDPN under flow.** A) Confluent human LECs were perfused with mouse blood for 4 min at 50 s^-1^ after treatment with vehicle (DMSO), 10 µM lotrafiban or 10 µM dasatinib. Slides were immunostained with anti-podoplanin (PDPN)/anti-rat Alexa-568 and anti-mouse CD41-FITC antibodies. Representative pictures are shown. B) LECs were perfused with blood from litter-matched CLEC-2^floxed/floxed;^ ^CreERT2^ mice treated with either tamoxifen to induce CLEC-2 deletion or corn oil as control (6 min, 50 s^-1^, two mice used per run). Samples were immunostained with anti-mouse CD41-PE and anti-podoplanin/anti-rat Alexa-488 antibodies. Representative pictures are shown. C) Percentage of platelet surface coverage is shown as mean ± SEM, n=3. *p<0.05 (significant differences vs corn oil-treated mice by two-tailed Student’s t-test). D) Platelet surface coverage quantification from experiments in (A) plotted as mean ± SEM, n=3. *p<0.05 (significant differences vs vehicle by one-way ANOVA and Dunnett’s post-test). E) Channels were coated with 100 µg/ml hFcPDPN and blocked with BSA. Heparinised blood treated with 5 µg/ml DiOC6 and either vehicle (DMSO) or 10 µM lotrafiban was perfused for 4 min at 50 s^-1^. Representative images are shown, n=3. Scale bars: 20 µm.

Human LECs were previously shown to induce activation and P-selectin exposure of wild-type (WT) mouse platelets *in vitro* (6, 29). These reports led us to test whether human LECs could support binding and aggregate formation of WT mouse platelets under flow. Similarly to human platelets, mouse platelets attached and aggregated on LECs at 50 s^-1^ (►Figure 3A). The αIIbβ3 blocker lotrafiban abrogated mouse platelet aggregation but had no effect on adhesion to LECs (►Figure 3D).

We took advantage of our observation to investigate if platelets could bind to LECs in the absence of CLEC-2 using a tamoxifen-inducible CLEC-2 transgenic mouse model. Complete loss of platelet CLEC-2 expression in the tamoxifen-treated mice but not in corn oil-injected control mice was confirmed by flow cytometry on the experimental day (Suppl. Figure 3, available online at www.thrombosis-online.com). Control and CLEC-2-deficient mice displayed comparable platelet counts (data not shown). While platelets from control mice adhered to LECs at 50 s^-1^, CLEC-2-deficient platelets were unable to bind to these cells. However, we observed binding to small gaps between LECs, showing that the platelets were susceptible to activation by the exposed matrix (►Figure 3B, C). These results confirm that CLEC-2 is the adhesive receptor which induces platelet arrest on podoplanin-expressing surfaces.

We then tested the ability of mouse platelets to bind to hFcPDPN at 50 s^-1^. Mouse platelets arrested and built aggregates onto hFcPDPN (►Figure 3E). Since mouse platelets do not express FcγRIIa, this result again showed the competency of hFcPDPN to bind flowing platelets independent of Fc binding.

### Optimal CLEC-2-mediated platelet binding to podoplanin under flow requires platelet intracellular signalling

**Figure 4:**
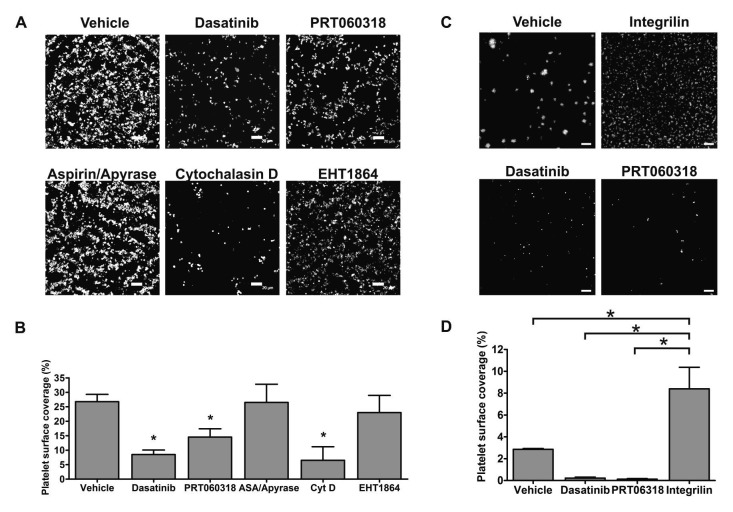
**Optimal CLEC-2 mediated platelet binding to podoplanin under flow requires platelet intracellular signalling.** A) Confluent human LECs were perfused with heparinised blood treated with Integrilin (9 µM) and either vehicle (DMSO), 10 µM dasatinib, 30 µM PRT060318, 1 mM aspirin (ASA) combined with 2 U/ml apyrase, 10 µM cytochalasin D (CytD) or the Rac inhibitor EHT1864 (100 µM) for 8 min at 50 s^-1^. Channels were stained for CD41. LEC monolayer integrity was confirmed by podoplanin immunostaining. Representative pictures are shown. B) Platelet surface coverage plotted as mean ± SEM, n=4. One-way ANOVA indicated a significant effect of treatments (p<0.005). *p<0.05 (significant differences vs vehicle-treated LECs by Dunnett’s post-test). C) Channels were coated with hFcPDPN and blocked with BSA. Heparinised blood treated with 5 µg/ml DiOC6, 10 µg/ml IV.3 antibody and either vehicle (DMSO), 9 µM Integrilin, 10 µM dasatinib or 30 µM PRT060318 was perfused for 4 min at 50 s^-1^. D) Quantification of platelet binding to hFcPDPN in the presence of the stated inhibitors (n=3). One-way ANOVA indicated a significant effect of treatments (p<0.005). *p<0.05 (significant differences vs binding in the presence of Integrilin after Dunnett’s post-test). Scale bars: 20 µm.

Syk mediates phosphorylation of the CLEC-2 hemITAM motif, with Src family kinases playing a critical role in CLEC-2-dependent signalling through the regulation of Syk and other effector proteins (22). To investigate whether inhibition of platelet intracellular signalling downstream of CLEC-2 affects platelet binding to LECs, we perfused human whole blood at 50 s^-1^ in the presence of different inhibitors reported to affect the CLEC-2 signalling cascade (30) together with Integrilin to exclusively assess platelet-LEC interactions. Neither the Rac inhibitor EHT1864 (100 µM) nor combined apyrase (2 U/ml) and aspirin (1 mM) to block secondary mediators reduced percentage of platelet surface coverage (►Figure 4A, B). Similarly, blockade of PLCγ2 using the nonspecific inhibitor U73122 did not reduce platelet adhesion to LECs (Suppl. Figure 4, available online at www.thrombosis-online.com). In contrast, the actin cytoskeleton blocker cytochalasin D or dasatinib (Sprycel), which inhibits Src kinases, greatly reduced platelet binding (►Figure 4A, B). Noteworthy, Src inhibition allowed a significantly lower platelet adhesion than αIIbβ3 blockade (►Figure 1A, B).

We also studied the role of the kinase Syk, a crucial component of the CLEC-2 signalling cascade present exclusively in haematopoietic cells. We first established the minimum concentration of the highly selectively Syk inhibitor PRT060318(31) required for complete Syk blockade in whole blood using an *in vitro* impedance aggregometry assay. As illustrated by Suppl. Figure 5 (available online at www.thrombosis-online.com), PRT060318 at 20 µM did not fully abrogate Syk-dependent collagen-induced aggregation, whereas full blockade was seen at 30 µM. At this concentration, PRT060318 diminished human platelet binding to LECs by 45% in the presence of Integrilin (►Figure 4B), further suggesting that platelet signalling supports adhesion to LECs.

Interestingly, fixation of LECs with 1% formalin did not abrogate platelet binding (Suppl. Figure 6, available online at www.thrombosis-online.com). Fixed LECs also supported platelet aggregate formation (data not shown). On the other hand, platelet binding was abolished when blood samples were formalin-fixed. These results are consistent with the notion that platelet but not LEC intracellular signalling contributes to platelet binding to LECs under flow.

To verify that intracellular signalling is required for optimal platelet binding to podoplanin, we investigated the effect of Src and Syk inhibitors on platelet adhesion to hFcPDPN (►Figure 4C). Dasatinib and PRT060318 significantly reduced platelet deposition to hFcPDPN (►Figure 4D), confirming that CLEC-2 mediated signalling enhances adhesion to podoplanin.

**Figure 5:**
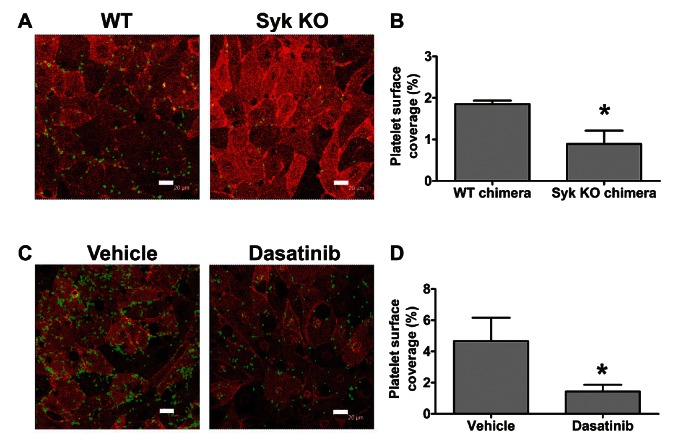
**Mouse platelet binding to LECs under flow requires Src and Syk.** Confluent human LECs were perfused for 4 min at 50 s^-1^ with heparinised mouse blood in the presence of 10 µM lotrafiban. Samples were immunostained with anti-podoplanin-PE and anti-mouse CD41-FITC. Representative pictures from (A) assays with WT vs Syk-deficient chimeric mice, n=5 and (C) assays comparing vehicle- (DMSO) vs 10 µM dasatinib-treated mouse blood, n=3. Scale bars: 20 µm. Percentage of platelet surface coverage is plotted as mean ± SEM. *p<0.05 (significant differences vs WT chimeras (B) or vehicle-treated blood (D) after one-tailed Student’s t-test).

To support the results obtained with PRT060318, we generated mouse radiation chimeras and compared the binding of WT and Syk-deficient mouse platelets to LECs (►Figure 5A). Lack of P-selectin exposure in response to CLEC-2 agonists in Syk-deficient platelets was confirmed by flow cytometry before the experiments (Suppl. Figure 7, available online at www.thrombosis-online.com). WT chimeras showed a 27% reduction in platelet count compared to non-irradiated WT control mice (data not shown), possibly due to suboptimal restoration of bone marrow function following reconstitution. As a consequence, the observed WT platelet adhesion was lower in this set of experiments. Although Syk-deficient chimeric mice only showed an additional 20% reduction in platelet count compared to WT chimeras, adhesion of Syk-deficient platelets was 50% lower than WT chimeric controls (►Figure 5B). These results demonstrated that Syk activity is required for optimal adhesion of flowing platelets to LECs. Noteworthy, treatment of human or mouse platelets with dasatinib similarly reduced the platelet surface area covering LECs under non-aggregating conditions (compare ►Figure 4A and ►Figure 5C). The inhibitory effect of dasatinib was also seen in the absence of Integrilin (►Figure 3A, D).

### Src/Syk-dependent platelet intracellular signalling stabilises platelet binding to podoplanin under flow

**Figure 6:**
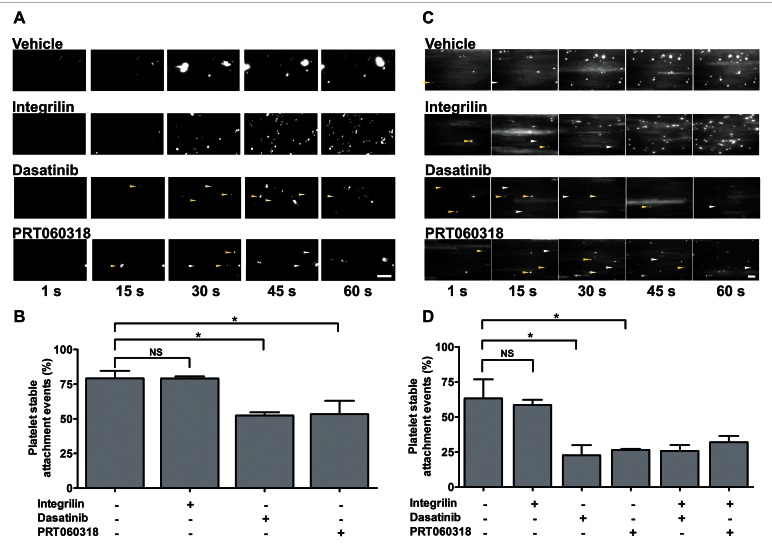
**Src/Syk dependent platelet intracellular signalling stabilises human platelet binding to podoplanin under flow.** Heparinised human blood was perfused in the presence of inhibitors or vehicle as appropriate (4 min, 50 s^-1^). A) Whole blood incubated with FITC-conjugated antibodies against CD41 and CD42b perfused on LECs. C) Whole blood incubated with DiOC6 (4 µM) and IV.3 antibody (10 µg/ml) perfused on hFcPDPN. Representative time-lapse images of platelet adhesion events are shown. Arrows indicate unstable platelet adhesions (yellow: attachment, white: detachment). Binding stability (measured as adhesion events lasting ≥ 10 s) on LECs (B) and hFcPDPN (D) in the presence of the stated inhibitors, mean ± SEM, n=3. One-way ANOVA indicated a significant effect of treatments (p<0.05). *p<0.05 (significant differences vs vehicle after Dunnett’s post-test). Scale bars: 20 µm.

In light of our results, we investigated whether platelet signalling through Src and Syk kinases is required to stabilise adhesion of flowing platelets to LECs. Human blood was incubated with FITC-conjugated anti-CD41 and CD42b antibodies and inhibitors or vehicle control as appropriate. Blood was perfused over LEC monolayers at 50 s^-1^ and time-lapse digital images collected (► Figure 6A and Suppl. Video 1, available online at www.thrombosis-online.com). Platelet binding stability, measured as percentage of adhesion events lasting ≥ 10 s, was evaluated off-line. In the absence of inhibitors, platelet adhesion to LECs involved initial rolling followed by stable binding in around 75% of events. Pre-treatment with Src or Syk inhibitors did not preclude platelet interaction with LECs but significantly decreased binding stability, making platelets more prone to dissociate from the LEC surface, while Integrilin did not modify adhesion stability (►Figure 6B and Suppl. Video 1, available online at www.thrombosis-online.com).

To verify whether Src and Syk kinases are also required to stabilize platelet adhesion to hFcPDPN under flow, we analysed binding of DiOC6-labelled platelets to hFcPDPN using time-lapse microscopy. Platelets often displayed transient rolling before becoming firmly adhered to hFcPDPN. In agreement with their effect on platelet binding to LECs, dasatinib and PRT060318 reduced binding stability to hFcPDPN (►Figure 6C and Suppl. Video 2, available online at www.thrombosis-online.com), although they displayed a stronger effect. In contrast, stability was not affected by Integrilin used alone, neither was further reduced when dasatinib or PRT060318 were combined with Integrilin (►Figure 6D), confirming that αIIbβ3 activation does not stabilise platelet adhesion to podoplanin.

## Discussion

It is well recognised that shear affects the binding capacity of adhesive receptors to their ligand pairs by influencing the efficiency of cell recruitment onto the surface and the lifetime of adhesive bonds once formed (32). While the adhesion mechanisms governing binding of platelets to immobilised extracellular matrix components under different shear conditions have been extensively studied (32), a comprehensive description of the conditions allowing blood platelets to arrest under flow onto a podoplanin-expressing surface was lacking. Podoplanin is expressed by multiple cell types including LECs, kidney podocytes, lung type I alveolar cells, fibroblastic reticular cells and the choroid plexus epithelium (33). Since the interaction between CLEC-2 and podoplanin is essential for lymphatic development and maintenance during adulthood (6, 14), we chose human LECs as a model to characterise binding of platelets to podoplanin.

Our data defined the ability of LECs to support human platelet attachment under flow. Subsequent aggregate formation was blocked by Integrilin. Inhibition of aggregation resulted in an elevated surface coverage relative to controls. This apparently paradoxical observation has been observed in previous platelet adhesion studies on collagen (34, 35). Since platelet stable adhesion to LECs leads to platelet aggregation, both podoplanin and activated αIIbβ3 integrins represent potential binding sites for subsequently perfused platelets. CLEC-2 levels in human platelets are much lower than those for integrin αIIbβ3 (2000 vs 64200–83300 per platelet, respectively) (36, 37). These expression differences probably explain why subsequently perfused platelets preferentially bind to activated αIIbβ3 in already bound platelets above available podoplanin sites. The lack of such competition against αIIbβ3 sites under non-aggregating conditions likely explains the observed significant increase in human platelet surface coverage on LECs in the presence of Integrilin. Additionally, this may reflect an increase in the number of platelet-podoplanin interactions taking place in the absence of large aggregates which would shield the podoplanin surface downstream of flow.

Although αIIbβ3 inhibitors similarly abrogated human and mouse aggregate formation on LECs, their effects on overall platelet adhesion were different between species. While human platelet surface coverage was significantly increased under non-aggregating conditions compared to vehicle control, lotrafiban did not modify overall adhesion of mouse platelets to LECs. This is likely explained by the preferential binding of lotrafiban-treated mouse platelets to LEC-LEC contacts, whereas Integrilin-treated human platelets were deposited as a homogeneous monolayer on the whole LEC surface. Multiple potential factors could have contributed to the observed interspecies differences, including a lower affinity of mouse CLEC-2 for human podoplanin leading to mouse platelet binding only in areas of high podoplanin density due to increased avidity. Noteworthy, our confocal microscopy often showed higher podoplanin staining in LEC-LEC contacts.

Platelets and LECs interacted efficiently at shear rates of 50 s^-1^, with significant binding still occurring at 150 s^-1^. These experimental conditions represent those found in the human venous circulation (24). Platelet binding was inefficient at 450 s^-1^ and totally absent at 1,350 s^-1^, indicating that arterial shear rates preclude platelet binding to podoplanin. Given than the average lymph flow/shear in the final lymphatic tracts are lower than those in the venous circulation into which they empty (38), our data confirm that platelets should be able to interact with podoplanin-expressing cells at the end of the thoracic duct (14).

Platelet adhesion to LECs was totally dependent on the presence of CLEC-2 and podoplanin, as binding was absent when CLEC-2-deficient blood or vascular endothelial cells lacking podoplanin were tested. We did not identify a role for CD31 or CD61 as facilitators of platelet binding to podoplanin. Although other potential interactions have not been ruled out, the ability of purified podoplanin and LECs to support similar platelet attachment indicates that CLEC-2 possesses intrinsic adhesive properties and that no other receptors are required to induce platelet arrest on podoplanin. Our results indicated that LECs promoted 1.5 times higher platelet binding than hFcPDPN (Suppl. Table 1, available online at www.thrombosis-online.com). This is likely due to differences in podoplanin density and mobility between the two surfaces. CLEC-2 binding to their ligands leads to receptor multimerisation (39), which in turn modifies podoplanin disposition on the LEC surface through lateral mobility leading to podoplanin clustering and more stable binding due to increased avidity (40). Although we have demonstrated the feasibility of CLEC-2 binding to immobilised hFcPDPN, the absence of lateral mobility under these conditions probably limited CLEC-2 clustering making platelet binding slightly less stable. The behaviour of platelets bound to hFcPDPN and LECs was comparable, with similar shear rates allowing binding to both surfaces.

A significant observation emerging from our data was that platelet signalling is required for stable adhesion to LECs. This analysis was supported by a) the inhibitory effect of pharmacological inhibitors blocking Src kinases, Syk activity, or cytoskeletal reorganisation on platelet binding to LECs, b) the absence of platelet binding upon platelet but not LEC fixation and c) the blocking effect of dasatinib and PRT060318 on platelet adhesion to recombinant hFcPDPN. Although both phosphorylation of CLEC-2 and aggregation in human platelets are dependent on PLCγ2 activity, secondary mediators release and Rac activation (30, 41), blockade of these signalling components did not affect adhesion under our experimental conditions. This is likely due to their later involvement during activation, further downstream of Syk and Src kinases. Real-time analysis of adhesion to LECs and hFcPDPN indicated that Syk/Src inhibition did not preclude platelet-podoplanin interactions but reduced the stability of binding. A potential explanation for this signalling requirement could be signalling-dependent receptor clustering leading to an increased avidity (40).

The use of Syk-deficient mice further demonstrated that absence of Syk significantly reduces platelet binding to LECs under flow. Unstable platelet adhesion leading to reduced platelet-LEC interactions likely contribute to the blood-lymphatic mixing and oedema observed in Syk-deficient mice, although additional mechanisms are expected to play a role. Several inhibitors against Syk are currently undergoing clinical trials against rheumatoid arthritis, asthma and immune thrombocytopenic purpura (42). Treatment with the Src/Abl inhibitor dasatinib, used against chronic myeloid leukaemia, can result in superficial oedema and pleural, pericardial or ascites effusions in a third of patients (43, 44). Consequently, understanding the physiological role played by Src and Syk in adult lymphatic maintenance, and how it is affected upon long-term pharmacological inhibition, represents an essential task.

Our results additionally indicate that physiologically relevant interactions between platelet CLEC-2 and other podoplanin-expressing cells might occur in the circulation. Expression of adhesion molecules in metastatic tumour cells allows their interaction with platelets and vascular endothelium, a crucial event to promote localized clotting activation and thrombus formation (45, 46). The capacity of podoplanin, up-regulated in metastatic tumours, to induce platelet activation and cancer cell arrest and extravasation (47, 48), makes tempting to speculate that CLEC-2-podoplanin interactions might play a role in cancer-associated venous thrombosis. Oscillatory low-average shear stress (<0.4 Pa), prevalent at arterial bends and bifurcations, stimulates an atherogenic phenotype (24). Given that the interaction between podoplanin-expressing activated macrophages and platelet CLEC-2 has been shown to induce platelet aggregation and degranulation (49), we hypothesize that this interaction might contribute to the thrombogenicity of ruptured atherosclerotic plaques containing podoplanin-expressing foam cells (50) in regions with disrupted flow (i. e. atherothrombosis).

In conclusion, this is the first detailed characterisation of the conditions allowing platelet CLEC-2 binding to podoplanin-expressing LECs under flow. Our data reveal for the first time that CLEC-2 possesses intrinsic adhesive properties and that subsequent platelet intracellular signalling through Src and Syk is required to stabilise CLEC-2-podoplanin interactions. In light of our observations we postulate that adhesive interactions between platelet CLEC-2 and podoplanin-expressing cells under venous shear might be physiologically important in the context of adult lymphatic maintenance, atherothrombosis, cancer-associated venous thrombosis and metastasis.

**What is known about this topic?**

- Podoplanin, the only known endogenous ligand for the platelet receptor CLEC-2, induces platelet activation and aggregation through Src and Syk kinases.
- Absence of platelet CLEC-2 or Syk, or lymphatic endothelial podoplanin in mice leads to blood-lymphatic mixing, suggesting that platelet binding to lymphatic endothelial cells and subsequent platelet activation are required for normal lymphatic development.
- Podoplanin and CLEC-2 are supposed to interact *in vivo* under conditions of low flow and low shear.

**What does this paper add?**

- This is the first detailed characterisation of the conditions allowing platelet CLEC-2 binding to podoplanin under flow.
- This is the first description of the intrinsic adhesive properties of the platelet receptor CLEC-2.
- This is the first report demonstrating that platelet intracellular signalling through Syk and Src kinases is required for stable platelet binding to podoplanin under flow.

## Supplementary Material

Suppl. Figures

Suppl. Video 1

Suppl. Video 1

## References

[1] Hägerling R Pollmann C Andreas M A novel multistep mechanism for initial lymphangiogenesis in mouse embryos based on ultramicroscopy. EMBO J 2013; 32: 629–644. 23299940 10.1038/emboj.2012.340 PMC3590982

[2] Yang Y Garcia-Verdugo JM Soriano-Navarro M Lymphatic endothelial progenitors bud from the cardinal vein and intersomitic vessels in mammalian embryos. Blood 2012; 120: 2340–2348. 22859612 10.1182/blood-2012-05-428607 PMC3447786

[3] Tober J Koniski A McGrath KE The megakaryocyte lineage originates from hemangioblast precursors and is an integral component both of primitive and of definitive hematopoiesis. Blood 2007;109: 1433–1441. 17062726 10.1182/blood-2006-06-031898 PMC1794060

[4] Bertozzi CC Schmaier AA Mericko P Platelets regulate lymphatic vascular development through CLEC-2-SLP-76 signaling. Blood 2010; 116: 661–670. 20363774 10.1182/blood-2010-02-270876 PMC3324297

[5] Bohmer R Neuhaus B Buhren S Regulation of developmental lymphangiogenesis by Syk(+) leukocytes. Dev Cell 2010; 18: 437–449. 20230750 10.1016/j.devcel.2010.01.009

[6] Finney BA Schweighoffer E Navarro-Núñez L CLEC-2 and Syk in the megakaryocytic/platelet lineage are essential for development. Blood 2012; 119: 1747–1756. 22186994 10.1182/blood-2011-09-380709 PMC3351942

[7] Ichise H Ichise T Ohtani O Phospholipase Cgamma2 is necessary for separation of blood and lymphatic vasculature in mice. Development 2009; 136: 191–195. 19056831 10.1242/dev.025353

[8] Schacht V Ramirez MI Hong YK T1alpha/podoplanin deficiency disrupts normal lymphatic vasculature formation and causes lymphedema. EMBO J 2003; 22: 3546–3556. 12853470 10.1093/emboj/cdg342 PMC165612

[9] Uhrin P Zaujec J Breuss JM Novel function for blood platelets and podoplanin in developmental separation of blood and lymphatic circulation. Blood 2010; 115: 3997–4005. 20110424 10.1182/blood-2009-04-216069

[10] Fu J Gerhardt H McDaniel JM Endothelial cell O-glycan deficiency causes blood/lymphatic misconnections and consequent fatty liver disease in mice. J Clin Invest 2008; 118: 3725–3737. 18924607 10.1172/JCI36077 PMC2567837

[11] Carramolino L Fuentes J Garcia-Andres C Platelets play an essential role in separating the blood and lymphatic vasculatures during embryonic angiogenesis. Circ Res 2010; 106: 1197–1201. 20203303 10.1161/CIRCRESAHA.110.218073

[12] Kiefer F Brumell J Al-Alawi N The Syk protein tyrosine kinase is essential for Fcgamma receptor signaling in macrophages and neutrophils. Mol Cell Biol 1998; 18: 4209–4220. 9632805 10.1128/mcb.18.7.4209 PMC109005

[13] El Zawahry MD Sayed NM El-Awady HM A study of the gross, microscopic and functional anatomy of the thoracic duct and the lympho-venous junction. Int Surg 1983; 68: 135–138. 6350208

[14] Hess PR Rawnsley DR Jakus Z Platelets mediate lymphovenous hemostasis to maintain blood-lymphatic separation throughout life. J Clin Invest 2014; 124: 273–284. 24292710 10.1172/JCI70422 PMC3871239

[15] Bender M May F Lorenz V Combined in vivo depletion of glycoprotein VI and C-type lectin-like receptor 2 severely compromises hemostasis and abrogates arterial thrombosis in mice. Arterioscler Thromb Vasc Biol 2013; 33: 926–934. 23448972 10.1161/ATVBAHA.112.300672 PMC6485540

[16] May F Hagedorn I Pleines I CLEC-2 is an essential platelet-activating receptor in hemostasis and thrombosis. Blood 2009; 114: 3464–3472. 19641185 10.1182/blood-2009-05-222273

[17] Andre P Morooka T Sim D Critical role for Syk in responses to vascular injury. Blood 2011; 118: 5000–5010. 21881044 10.1182/blood-2011-06-360743 PMC3208305

[18] Law DA Nannizzi-Alaimo L Ministri K Genetic and pharmacological analyses of Syk function in αIIbβ3 signaling in platelets. Blood 1999; 93: 2645–2652. 10194444

[19] Pan Y Yago T Fu J Podoplanin requires sialylated O-glycans for stable expression on lymphatic endothelial cells and for interaction with platelets. Blood 2014; 124: 3656–3665. 25336627 10.1182/blood-2014-04-572107 PMC4256915

[20] Hughes CE Navarro-Núñez L Finney BA CLEC-2 is not required for platelet aggregation at arteriolar shear. J Thromb Haemost 2010; 8: 2328–2332. 20695981 10.1111/j.1538-7836.2010.04006.x PMC4362701

[21] Ventura A Kirsch DG McLaughlin ME Restoration of p53 function leads to tumour regression in vivo. Nature 2007; 445: 661–665. 17251932 10.1038/nature05541

[22] Séverin S Pollitt AY Navarro-Núñez L Syk-dependent phosphorylation of CLEC-2: a novel mechanism of hem-immunoreceptor tyrosine-based activation motif signaling. J Biol Chem 2011; 286: 4107–4116. 21098033 10.1074/jbc.M110.167502 PMC3039337

[23] Turner M Mee PJ Costello PS Perinatal lethality and blocked B-cell development in mice lacking the tyrosine kinase Syk. Nature 1995; 378: 298–302. 7477352 10.1038/378298a0

[24] Malek AM Alper SL Izumo S. Hemodynamic shear stress and its role in atherosclerosis. J Am Med Assoc 1999; 282: 2035–2042. 10.1001/jama.282.21.2035 10591386

[25] Schneider CA Rasband WS Eliceiri KW NIH Image to ImageJ: 25 years of image analysis. Nat Methods 2012; 9: 671–675. 22930834 10.1038/nmeth.2089 PMC5554542

[26] Bazzoni G Dejana E. Endothelial cell-to-cell junctions: molecular organisation and role in vascular homeostasis. Physiol Rev 2004; 84: 869–901. 15269339 10.1152/physrev.00035.2003

[27] Huang J Roth R Heuser JE Integrin alpha(v)beta(3) on human endothelial cells binds von Willebrand factor strings under fluid shear stress. Blood 2009; 113: 1589–1597. 18927433 10.1182/blood-2008-05-158584 PMC2644087

[28] Mangin P Yuan Y Goncalves I Signaling role for phospholipase C gamma 2 in platelet glycoprotein Ib alpha calcium flux and cytoskeletal reorganisation. Involvement of a pathway distinct from FcR gamma chain and Fc gamma RIIA. J Biol Chem 2003; 278: 32880–32891. 12813055 10.1074/jbc.M302333200

[29] Borgognone A Navarro-Núñez L Correia JN CLEC-2-dependent activation of mouse platelets is weakly inhibited by cAMP but not by cGMP. J Thromb Haemost 2014; 12: 550–559. 24460629 10.1111/jth.12514 PMC4138994

[30] Pollitt AY Grygielska B Leblond B Phosphorylation of CLEC-2 is dependent on lipid rafts, actin polymerisation, secondary mediators, and Rac. Blood 2010; 115: 2938–2946. 20154214 10.1182/blood-2009-12-257212

[31] Reilly MP Sinha U Andre P PRT-060318, a novel Syk inhibitor, prevents heparin-induced thrombocytopenia and thrombosis in a transgenic mouse model. Blood 2011; 117: 2241–2246. 21088136 10.1182/blood-2010-03-274969 PMC3568699

[32] Ruggeri ZM Mendolicchio GL Adhesion mechanisms in platelet function. Circ Res 2007; 100: 1673–1685. 17585075 10.1161/01.RES.0000267878.97021.ab

[33] Navarro-Núñez L Langan SA Nash GB The physiological and pathophysiological roles of platelet CLEC-2. Thromb Haemost 2013; 109: 991–998. 23572154 10.1160/TH13-01-0060 PMC3693086

[34] Auger JM Kuijpers MJ Senis YA Adhesion of human and mouse platelets to collagen under shear: a unifying model. FASEB J 2005;19: 825–827. 15758040 10.1096/fj.04-1940fje

[35] Melis E Bonnefoy A Daenens K αIIbβ3 antagonism vs. antiadhesive treatment to prevent platelet interactions with vascular subendothelium. J Thromb Haemost 2004; 2: 993–1002. 15140136 10.1111/j.1538-7836.2004.00747.x

[36] Burkhart JM Vaudel M Gambaryan S The first comprehensive and quantitative analysis of human platelet protein composition allows the comparative analysis of structural and functional pathways. Blood 2012; 120: e73-e82. 22869793 10.1182/blood-2012-04-416594

[37] Gitz E Pollitt AY Gitz-Francois JJ CLEC-2 expression is maintained on activated platelets and on platelet microparticles. Blood 2014; 124: 2262–2270. 25150298 10.1182/blood-2014-05-572818 PMC4183985

[38] Zawieja DC Contractile physiology of lymphatics. Lymphat Res Biol 2009; 7: 87–96. 19534632 10.1089/lrb.2009.0007 PMC2925033

[39] Hughes CE Pollitt AY Mori J CLEC-2 activates Syk through dimerisation. Blood 2010; 115: 2947–2955. 20154219 10.1182/blood-2009-08-237834 PMC4361903

[40] Pollitt AY Poulter NS Gitz E Syk and Src family kinases regulate CLEC-2 mediated clustering of podoplanin and platelet adhesion to lymphatic endothelial cells. J Biol Chem 2014; 289: 35695-35710. 25368330 10.1074/jbc.M114.584284 PMC4276840

[41] Suzuki-Inoue K Fuller GL Garcia A A novel Syk-dependent mechanism of platelet activation by the C-type lectin receptor CLEC-2. Blood 2006; 107: 542–549. 16174766 10.1182/blood-2005-05-1994

[42] Kaur M Singh M Silakari O. Inhibitors of switch kinase ‘spleen tyrosine kinase’ in inflammation and immune-mediated disorders: A review. Eur J Med Chem 2013; 67: 434–446. 23917087 10.1016/j.ejmech.2013.04.070

[43] Goldblatt M Huggins JT Doelken P Dasatinib-induced pleural effusions: a lymphatic network disorder? Am J Med Sci 2009; 338: 414–417. 19838099 10.1097/MAJ.0b013e3181ae9227

[44] Hochhaus A Kantarjian H. The development of dasatinib as a treatment for chronic myeloid leukemia (CML): from initial studies to application in newly diagnosed patients. J Cancer Res Clin Oncol 2013; 139: 1971–1984. 23942795 10.1007/s00432-013-1488-z PMC3825579

[45] Falanga A Russo L Verzeroli C. Mechanisms of thrombosis in cancer. Thromb Res 2013; 131 (Suppl 1): S59-S62. 23452745 10.1016/S0049-3848(13)70024-0

[46] Konstantopoulos K Thomas SN Cancer cells in transit: the vascular interactions of tumor cells. Annu Rev Biomed Eng 2009; 11: 177–202. 19413512 10.1146/annurev-bioeng-061008-124949

[47] Cueni LN Hegyi I Shin JW Tumor lymphangiogenesis and metastasis to lymph nodes induced by cancer cell expression of podoplanin. Am J Pathol 2010; 177: 1004–1016. 20616339 10.2353/ajpath.2010.090703 PMC2913355

[48] Kunita A Kashima TG Morishita Y The platelet aggregation-inducing factor aggrus/podoplanin promotes pulmonary metastasis. Am J Pathol 2007; 170: 1337–1347. 17392172 10.2353/ajpath.2007.060790 PMC1829466

[49] Kerrigan AM Navarro-Núñez L Pyz E Podoplanin-expressing inflammatory macrophages activate murine platelets via CLEC-2. J Thromb Haemost 2012; 10: 484–486. 22212362 10.1111/j.1538-7836.2011.04614.x PMC3433653

[50] Hatakeyama K Kaneko MK Kato Y Podoplanin expression in advanced atherosclerotic lesions of human aortas. Thromb Res 2012; 129: e70-e76. 22283975 10.1016/j.thromres.2012.01.003

