# Supplementary material for: Platelet Adhesion to Podoplanin Under Flow is Mediated by the Receptor CLEC-2 and Stabilised by Src/Syk-Dependent Platelet Signalling
Source: Thromb Haemost. 2015 Feb 19;113(5):1109–20. doi: 10.1160/TH14-09-0762 (PMC4990172; doi:10.1160/TH14-09-0762)
Supplement: Suppl. Figures [file TH-113-05-14090762-s001.pdf]

Supplemental Material to Navarro-Núñez et al. "Platelet adhesion to podoplanin under flow is mediated by the receptor CLEC-2 and stabilised by Src/Syk-dependent platelet signalling" (Thromb Haemost 2015; 113.5)

Suppl. Methods

Materials

Antibody sources were as follows: FITC- and PE- anti-mouse CD41 (clone MWReg30) from BD Pharmingen (San Diego, CA); anti-human CD41-FITC (clone HIP8) and CD42b-FITC (clone HIP1) from eBioscience (Hatfield, UK); mouse anti-human CD41-FITC (clone 5B12) from Dako (Ely, UK); anti-human podoplanin antibody NZ-1.3 from eBioscience (Hatfield, UK); Alexa Fluor 488- and 568-conjugated antibodies from Invitrogen (Paisley, UK); IV.3 antibody from StemCell Technologies (Manchester, UK); FITC-conjugated rat α-mouse P-selectin (clone Wug.E9) and rat IgG control from Emfret Analytics (Würzburg, Germany).

Rat anti-mouse CLEC-2 IgG antibody, kindly provided by Dr. Caetano Reis e Sousa, or a rat IgG2b isotype control (MAB0061, R&D Systems, Abingdon, UK) were conjugated with Alexa Fluor 488 using an Invitrogen kit (Paisley, UK) following the manufacturer's instructions. The blocking antibodies against CD61 (clone SZ21) and CD31 (clone YR-131-12) were from previously described sources (1, 2).

Lotrafiban was from GlaxoSmithKline (King of Prussia, PA) and Integrilin (Eptifibatide) from the University Hospital Birmingham's pharmacy. The Src kinase inhibitor dasatinib (Sprycel) was from LC Laboratories (Woburn, MA). The selective Syk inhibitor, PRT060318, was kindly donated by Portola Pharmaceuticals. Horm collagen was from Nycomed (Munich, Germany). Heparin was from Leo Pharma (Ballerup, Denmark). D-Phe-Pro-Arg-chloromethylketone-HCl (PPACK) was from BioVision (Milpitas, CA). The Rac inhibitor EHT1864 was from ExonHit Therapeutics (Paris, France). U73122 was purchased from Cayman Chemical (Ann Arbor, MI). Dr Johannes Eble provided the snake venom toxin rhodocytin. All other reagents were from Sigma-Aldrich (Poole, UK).

Human LECs were purchased from Promocell (Heidelberg, Germany) and cultured in the supplier's recommended medium (MV2) containing penicillin, streptomycin and gentamycin. Human umbilical vein endothelial cells (HUVECs) were isolated from umbilical cords obtained after informed consent from the University of Birmingham Human Biomaterials Resource Centre (ethical approval reference 09/H1010/75), and cultured as described elsewhere (3).

The extracellular domain of human podoplanin (amino acids 23–131) was amplified from human dermal microvascular endothelial cells cDNA with primers hPodoF*Hind*III (GTCAGCAGGAAGCTTCCAGGAGAGCAACAACTCAAC) and hPodoR*Bam*HI (TCGGCTCCGGATCCACTGTTGACAAACCATCTTTCTC). After digestion with HindIII and BamHI the product was cloned into IgFc vector pcDNA3Ig to yield a construct encoding the podoplanin extracellular domain fused at the COOH terminus to the Fc region of human IgG1. Recombinant protein was expressed in human 293T cells and purified as previously described (4).

Whole blood impedance aggregometry

Heparinized human blood was diluted 1:1 using saline (0.9%) and stirred for 3 minutes at 37°C. PRT060318 at the stated concentrations or DMSO as control were pre-incubated at 37°C for 1 minutes. Following collagen stimulation (10 μg/ml), aggregation was monitored at 37°C for 6 minutes, calculated by impedance across two electrode pairs (Multiplate analyser, Instrumentation Laboratory UK Ltd, Warrington, UK), and reported in arbitrary impedance units (AU).

Flow cytometry:

Mouse CLEC-2 was detected in blood diluted 1:10 with Tyrode's buffer using Alexa Fluor 488-conjugated rat anti-mouse CLEC-2 or rat IgG2b isotype control antibodies. For the detection of Syk-dependent P-selectin exposure, mouse whole blood was stimulated with 100 nM rhodocytin for 5 minutes at 37°C in the presence of 5 μl FITC–labelled anti-mouse P-selectin. Non-stimulated platelets were used as negative control. Expression of human podoplanin was assessed in HUVECs and LECs using a phycoerythrin-conjugated rat anti-human podoplanin antibody. Endothelial cells incubated with a phycoerythrin-conjugated rat IgG2a isotype control were used as negative control. Cells were identified by their FSC/SSC profile in a FACScalibur flow cytometer using Cell-Quest software (BD Biosciences, Oxford, UK).

Suppl. Results

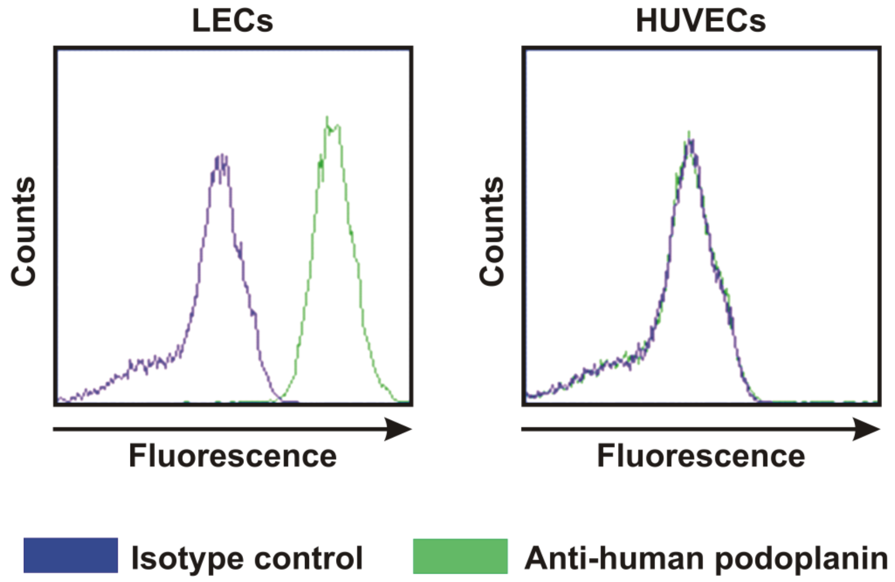

Suppl. Figure 1: Analysis of podoplanin expression in LECs and HUVECs by flow cytometry. Human podoplanin expression was assessed in human LECs and HUVECs by flow cytometry using a phycoerythrin-conjugated rat anti-human podoplanin antibody (green traces). Endothelial cells incubated with a phycoerythrin-conjugated rat IgG2a isotype control were used as negative control (violet traces). Cells were identified by their FSC/SSC profile. A representative example is shown.

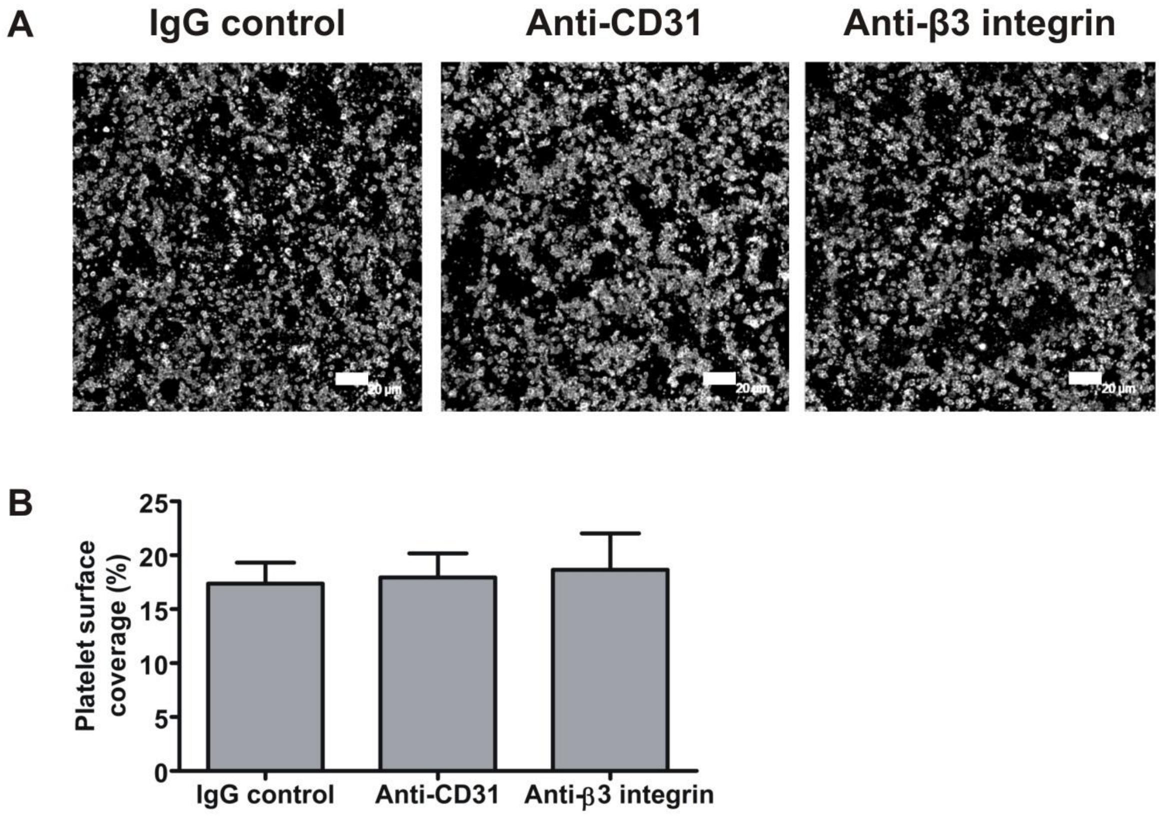

Suppl. Figure 2: CD31 or CD61 blocking antibodies do not affect human platelet binding to LECs. Confluent human LECs were treated for 10 minutes with either control IgG, anti-CD31 or anti-β3 integrin blocking antibodies (10 µg/ml), washed and then perfused for 8 minutes at 50 s^{-1} with heparinized whole blood in the presence of integrilin. Channels were washed, fixed and stained with anti-CD41/anti-mouse Alexa-488 antibodies. LEC monolayer integrity was confirmed by immunostaining for podoplanin. (A) Representative pictures are shown. Bars represent 20 µm. (B) Quantification of percentage of surface coverage from 3 independent experiments. Error bars represent SEM. No significant differences between treatments were found by one-way ANOVA test.

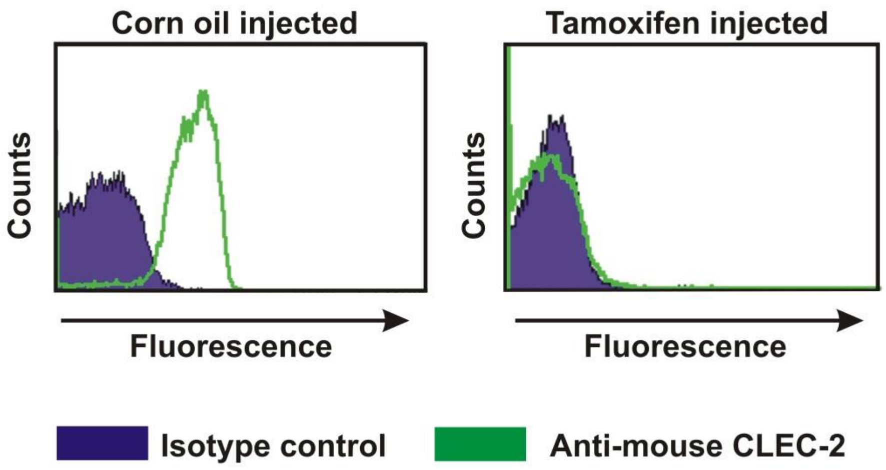

Suppl. Figure 3: Verification of CLEC-2 genotypes by flow cytometry. Platelet CLEC-2 expression in CLEC-2$^{floxed/floxed; CreERT2 mice}$ injected with tamoxifen to induce CLEC-2 excision or with corn oil as control was analysed in whole blood by flow cytometry using an Alexa Fluor 488-conjugated rat anti-mouse CLEC-2 (green traces). Platelets incubated with a rat IgG2b isotype control were used as negative control (filled violet traces). Platelets were identified by their FSC/SSC profile. A representative example from 3 independent experiments is shown.

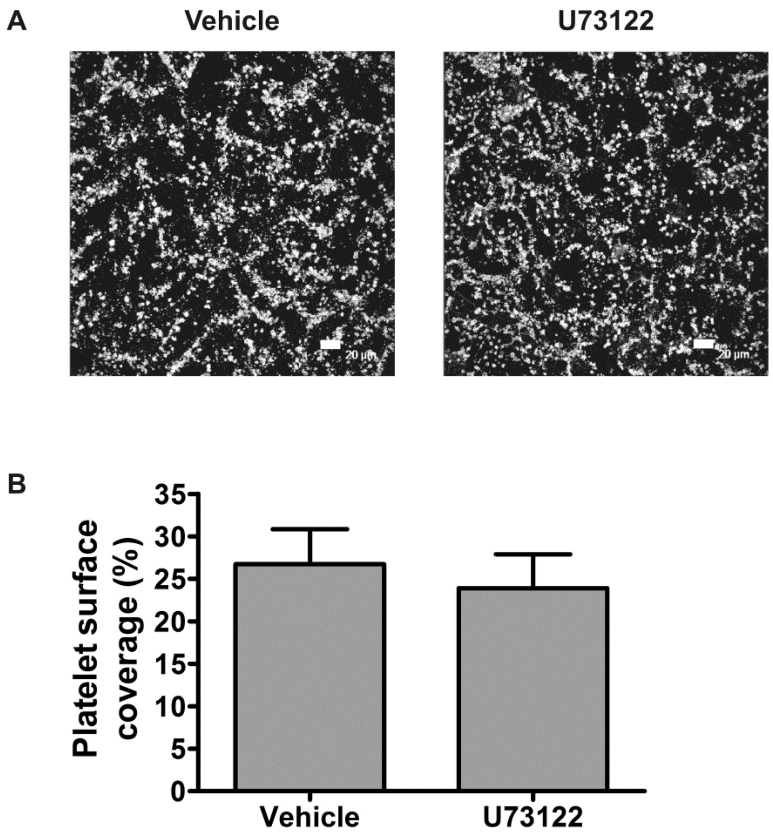

Suppl. Figure 4: PLCγ inhibition does not affect human platelet binding to LECs. Confluent human LECs were treated for 10 minutes with either vehicle control (DMSO) or the non-specific inhibitor of phospholipase C gamma (PLCγ) U73122 (10 μM) and then perfused for 8 minutes at 50 s^{-1} with heparinized whole blood in the presence of 9 μM integrilin. Channels were washed, fixed and stained with anti-CD41/anti-mouse Alexa-488 antibodies. LEC monolayer integrity was confirmed by immunostaining for podoplanin. (A) Representative pictures are shown. Bars represent 20 μm. (B) Quantification of percentage of surface coverage from 3 independent experiments. Error bars represent SEM. No significant differences between treatments were found by two-tailed paired t-test.

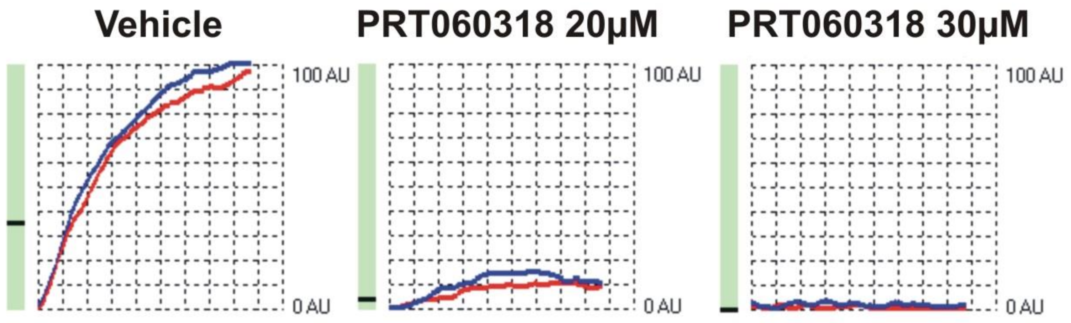

Suppl. Figure 5: Inhibition of collagen-induced whole blood platelet aggregation by PRT060318. Heparinized human blood was incubated with PRT060318 at the stated concentrations or DMSO vehicle control and then stimulated with 10 μg/ml collagen. Aggregation was monitored at 37°C for 6 min, calculated by impedance across two electrode pairs (red and blue lines) using a Multiplate analyser, and reported in arbitrary impedance units (AU).

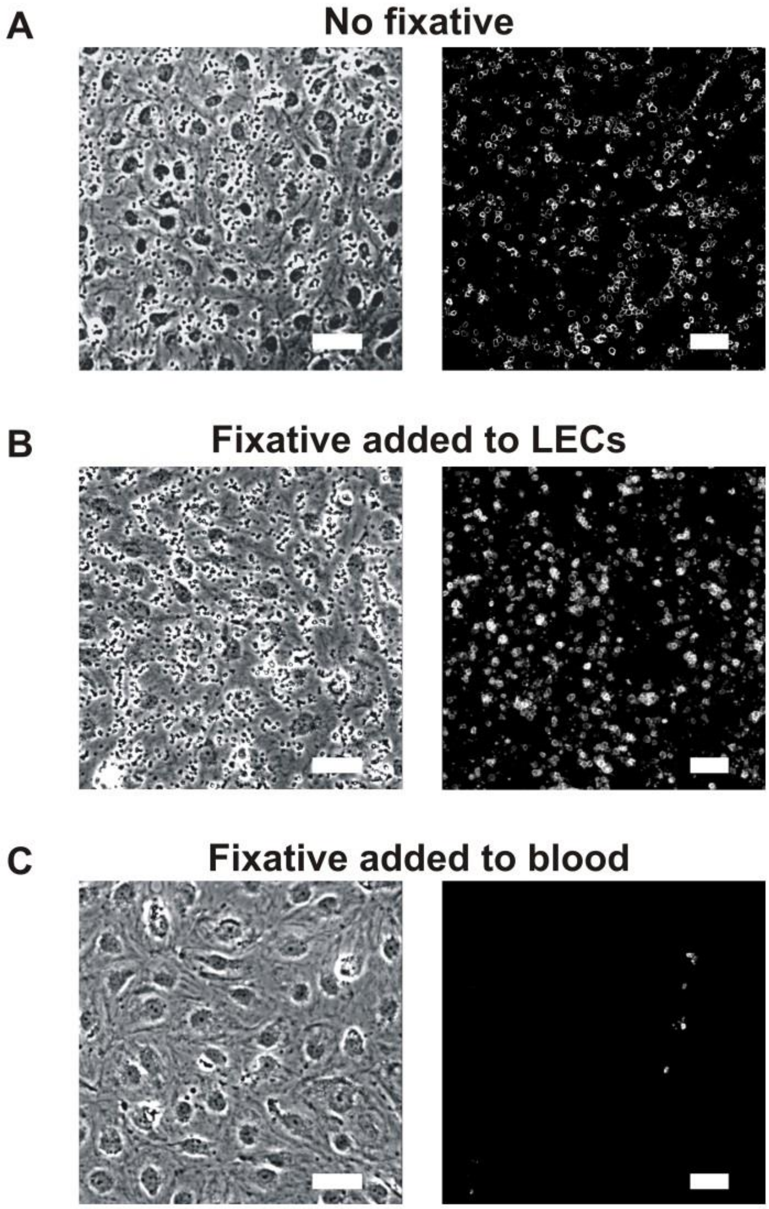

Suppl. Figure 6: Fixation of platelets but not LECs abrogates platelet binding to LEC monolayers. Confluent human LECs were treated for 15 minutes with either (A & C) PBS as control or (B) 1% formalin, washed and then perfused for 8 minutes at 50 s^{-1} with (A & B) unfixed or (C) 1% formalin-fixed heparinized blood in the presence of integrilin. Phase contrast images were obtained using a 20x lens, bars represent 100 μm. Fluorescence images were obtained in a different field of view using a 63x lens. Bars represent 20 μm. Each experiment was repeated twice.

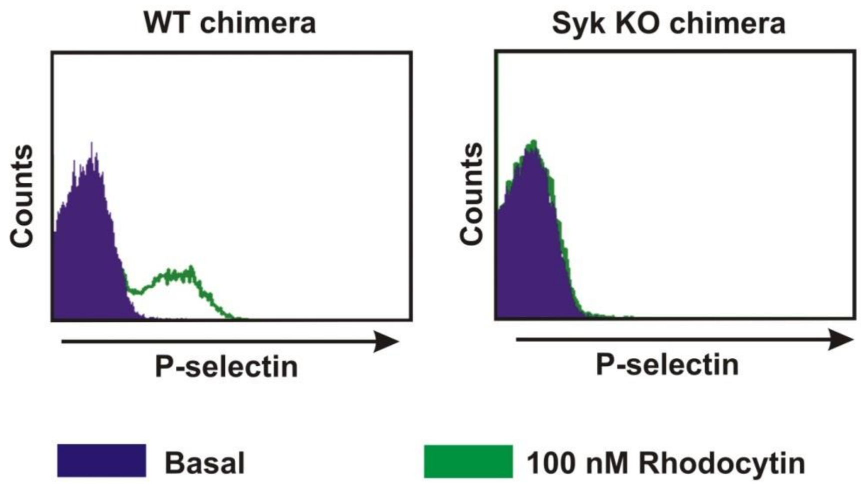

Suppl. Figure 7: Verification of chimeric mice genotypes by flow cytometry. Syk-dependent platelet P-selectin exposure was analysed in whole blood from WT or Syk-deficient chimeric mice. Blood was stimulated with 100 nM rhodocytin in the presence of FITC–labelled anti-mouse P-selectin antibody (green traces). Non-stimulated platelets were used as negative control (filled violet traces). Platelets were identified by their FSC/SSC profile. A representative example from 5 independent experiments is shown.

Suppl. Table 1: Comparison of platelet surface coverage on LECs and hFcPDPN at 50 s^{-1} under aggregating and non-aggregating conditions presented in the manuscript:

% Platelet binding	LECs	hFcPDPN	Comments
**Vehicle**	8.2% (Fig 1B, 8 min flow)	2.8% (Fig 2D, 4 min flow)	Binding to LECs 2.9 times higher with twice as many platelets perfused
**Integrilin**	27.9% (Fig 1B, 8 min flow)	8.4% (Fig 2D, 4 min flow)	Binding to LECs 3.3 times higher with twice as many platelets perfused

% Platelet binding per ml blood perfused	LECs	hFcPDPN	Comments
**Vehicle**	3.4%	2.3%	Binding to LECs 1.5 times higher under comparable conditions
**Integrilin**	11.6%	7%	Binding to LECs 1.6 times higher under comparable conditions

Suppl. Video 1: Src and Syk inhibitors reduce stability of platelet adhesion to LECs. To analyse real-time adhesion of platelets to LECs, heparinized whole blood was incubated with FITC-conjugated anti-human CD41 and CD42b antibodies at 1/100 dilution and inhibitors or vehicle control as appropriate for 10 minutes and then perfused over LEC monolayers at 50 s^{-1}. Digital images were captured at 500 ms interval with 500 ms exposure time and 2x2 binning settings to create 1 minute videos. Upper left: vehicle; upper right: 9 μM integrilin; lower left: 10 μM dasatinib; lower right: 30 μM PRT060318. See separate video file online at www.thrombosis-online.com.

Suppl. Video 2: Src and Syk inhibitors reduce stability of platelet adhesion to hFcPDPN. To analyse real-time adhesion of platelets to hFcPDPN, heparinized whole blood was incubated with 4 μM DiOC6, 10μg/ml IV.3 antibody and inhibitors or vehicle control as appropriate for 10 minutes and then perfused over hFcPDPN-coated channels at 50 s^{-1}. Digital images were captured at 250 ms interval with 200 ms exposure time and 2x2 binning settings to create 1 minute videos. Upper left: vehicle; upper right: 9 μM integrilin; lower left: 10 μM dasatinib; lower right: 30 μM PRT060318. See separate video file online at www.thrombosis-online.com.

Supplemental references:

1. Burton VJ, Butler LM, McGettrick HM, et al. Delay of migrating leukocytes by the basement membrane deposited by endothelial cells in long-term culture. Exp Cell Res 2011; 317: 276-292.
2. Diapouli FM. Investigating the reverse transmigration of neutrophils in human and murine in vitro models of inflammation. University of Birmingham eTheses Repository 2011; http://etheses.bham.ac.uk/1683.
3. Cooke BM, Usami S, Perry I, et al. A simplified method for culture of endothelial cells and analysis of adhesion of blood cells under conditions of flow. Microvasc Res 1993; 45: 33-45.
4. Schacht V, Dadras SS, Johnson LA, et al. Up-regulation of the lymphatic marker podoplanin, a mucin-type transmembrane glycoprotein, in human squamous cell carcinomas and germ cell tumors. Am J Pathol 2005; 166: 913-921.
